# Epiphyseal displacement of the distal humerus in a neonate: a case report

**DOI:** 10.31744/einstein_journal/2024RC0868

**Published:** 2024-11-22

**Authors:** Talissa Oliveira Generoso, Susana dos Reis Braga, Francesco Camara Blumetti, Maurício Pegoraro, Amancio Ramalho

**Affiliations:** 1 Hospital Israelita Albert Einstein São Paulo SP Brazil Hospital Israelita Albert Einstein, São Paulo, SP, Brazil.; 2 Instituto Brasil de Tecnologias da Saúde Rio de Janeiro RJ Brazil Instituto Brasil de Tecnologias da Saúde, Rio de Janeiro, RJ, Brazil.; 3 Santa Casa de Misericórdia de São Paulo São Paulo SP Brazil Santa Casa de Misericórdia de São Paulo, São Paulo, SP, Brazil.

**Keywords:** Epiphysis, Humerus, Birth injuries, Joint dislocations, Infant, newborn

## Abstract

Epiphyseal displacement of the distal humerus is rare and difficult to diagnose. In addition, the literature on the prognosis and treatment is limited. Here, we present a case of distal humeral physeal separation with significant displacement in a neonate. A favorable outcome was obtained following closed reduction and percutaneous fixation with Kirschner wires assisted by arthrography. This report adds valuable information on this subject to the existing literature.

## INTRODUCTION

Distal humeral physeal separations are rare injuries with a higher incidence in children <3 years of age, accounting for 7% of distal humeral injuries.^(1)^ In neonates, the mechanism of birth-related trauma typically involves rotational shear forces; however, it is essential to consider the possibility of non-accidental trauma.^(2–5)^

Due to age-related limitations, obtaining a comprehensive medical history and physical examination can make the diagnosis challenging. During this stage of development, the elbow bone epiphyses remain cartilaginous and physeal separations are difficult to visualize on radiographs, leading to diagnostic errors and delays. In such cases, elbow dislocation and lateral condylar fractures have been reported as the initial misdiagnoses.^(1,3,4,6)^

Due to the infrequent nature of these injuries, there is limited data in the literature regarding prognosis and treatment. Varus deformity is the most frequently reported complication, and anatomical reduction is essential for prevention. Some studies have suggested accepting the initial deformities in these fractures because of the high remodeling rate in children.^(6)^

Considering the scarcity of data on this topic and with the aim of contributing to the existing literature, we present a case report of a neonate with distal humeral physeal separation treated in our facility through closed reduction and percutaneous fixation with metallic wires.

## CASE REPORT

**Figure 1:**
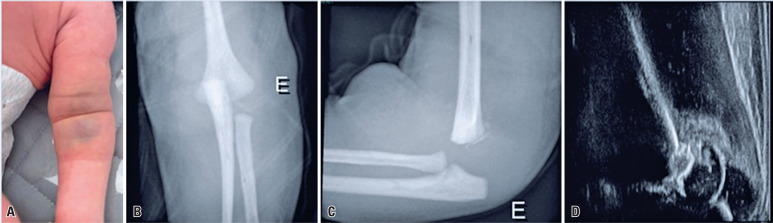
A) Clinical aspect of the left elbow; B and C) X-rays in the anterior–posterior and lateral view; D) ultrasound of the left elbow

A 9-day-old female patient with a cephalic presentation was delivered via cesarean section at full term. According to the parents, she had been experiencing left elbow edema and limited limb movement for the past 4 days. Upon physical examination, edema and bruising were observed, along with a reduced range of elbow flexion and extension, with an intact palmar grip (Figure 1A).

Radiography revealed posterior displacement of the ulnar diaphysis relative to the humerus and a small bony fragment in the distal humerus (Figure 1B and C). Ultrasonography confirmed distal humeral epiphyseal separation (Figure 1D).

**Figure 2:**
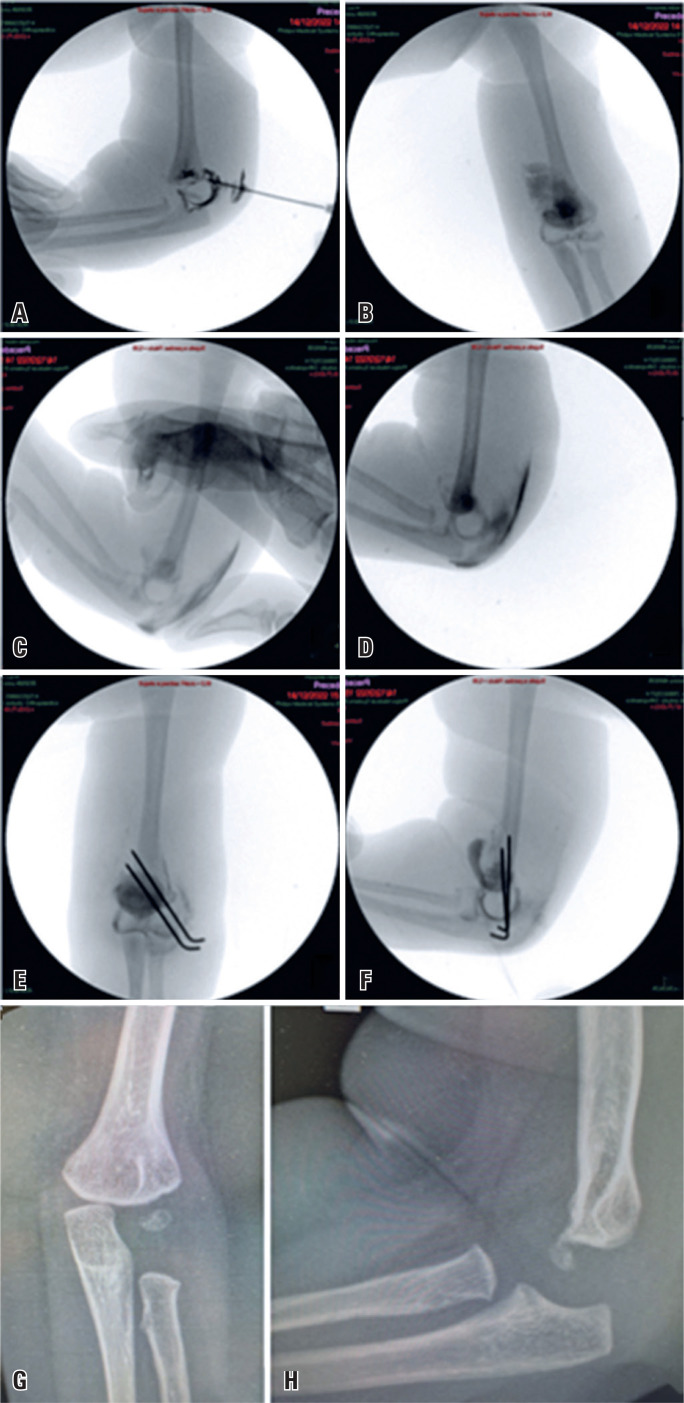
A and B) Assessment of the epiphyseal separation with the aid of an arthrogram; C) Successful closed reduction achieved with the elbow flexion maneuver and posterior support on the fragment; D) Loss of reduction upon elbow extension; E and F) Percutaneous fixation with two parallel lateral Kirschner wires; G and H) Radiographic images demonstrating consolidation and good alignment at 12 months after the injury

Due to the limited range of motion and significant fragment displacement, closed reduction was selected as the treatment approach. The patient was administered general anesthesia, and the fragment was identified using an arthrogram (Figure 2A and B). Anatomical reduction was achieved by flexing the elbow while providing posterior support to the fragment (Figure 2C); however, this reduction was lost during elbow extension (Figure 2D). Due to the instability observed during surgery, percutaneous fixation with two parallel 1 mm Kirschner wires was performed, providing satisfactory stability (Figure 2E and F).

Postoperatively, the cast splint was maintained for 2 weeks, after which the splint and wires were removed. One month after surgery, clinical improvement was observed with partial recovery of flexion and extension. By 6 weeks, active movement of the entire left upper limb was observed. After 12 months of follow-up, the patient exhibited a full range of elbow motion without angular deformities or an apparent length discrepancy, and the radiographs showed total consolidation and excellent bone alignment (Figure 2G and H).

This study was approved by the Ethics Committee of *Hospital Israelita Albert Einstein* (CAAE: 72484323.1.0000.0071; # 6.272.210).

## DISCUSSION

Elbow injuries are among the most common causes of emergency department visits in children; however, distal humeral physeal separation is rare and challenging to diagnose.

Considering that the secondary ossification centers of the elbow are not visible on radiographic examinations until approximately 8 months of age, diagnostic delays are quite common. They can range from 2 to 30 days after the injury,^(1,6)^ with the initial radiographic diagnosis absent in up to 56% of cases.^(5)^ Therefore, a high level of diagnostic suspicion should be maintained, and radiographic analysis should consider indirect signs, such as the fat pad sign, misalignment of the ulnar diaphysis with the humeral diaphysis on the anteroposterior view, and the relationship of the olecranon with the humeral diaphysis on the lateral view. Posteromedial displacement and reduced space between the proximal radial metaphysis and the anterior humeral line compared to the contralateral side were the most frequent signs.^(1,4,5)^ Additional imaging studies, such as ultrasonography, magnetic resonance imaging, and arthrography, may be required for a more comprehensive assessment.^(1,5)^ Intraoperatively, positioning the ulnar axis between the medial and lateral humeral lines on an anteroposterior radiograph can help guide proper reduction.^(7)^

Regarding the prognosis and treatment, injuries affecting the epiphyses and epiphyseal plates can lead to growth disturbances and deformities in the affected area. Cubitus varus is the most common complication of these injuries in children under 2 years, affecting 12.5-30% of cases.^(4,5)^ De Jager et al recommended closed reduction and percutaneous fixation in children <2 years of age.^(4)^ The literature also advises against late manipulation of these injuries, especially 4–7 days after trauma,^(1)^ to avoid further physeal damage. When the patient presented to our office, the timing of the injury was uncertain but was likely within 7 days based on the clinical signs. Therefore, a reduction was performed.

Cha et al. also recommended closed reduction and percutaneous fixation with arthrographic guidance, emphasizing anatomical reduction, especially in preschool children, as essential factors for preventing cubitus varus.^(8)^ This option was adopted in the present case.

Skaggs and Frick pointed out that closed reduction followed by immobilization may not be sufficient to prevent cubitus varus, and therefore recommended percutaneous fixation. They also stated that fractures diagnosed 7–10 days after trauma should not be manipulated to avoid further injury.^(3)^

In contrast, Jacobsen et al.^(6)^ argued that varus deformity is rare in neonates and suggested that reduction may not be necessary in this age group because of the favorable prognosis.

In this case, we initially chose closed reduction aided by arthrography because of the significant displacement. However, the instability observed during the procedure indicated the need for percutaneous fixation for added stability. Given the limited literature available, we believe that this approach yields satisfactory results.

The limitations of this study include the fact that it was a single case report with a short follow-up period.

## CONCLUSION

Distal humeral physeal separations are rare and challenging to diagnose. We present a case of significant displacement in a neonate whose outcome was satisfactory following closed reduction and percutaneous fixation with the aid of an arthrogram. We hope that this study adds valuable information to the existing literature.
